# Emissions from thaw ponds largely offset the carbon sink of northern permafrost wetlands

**DOI:** 10.1038/s41598-018-27770-x

**Published:** 2018-06-22

**Authors:** McKenzie Kuhn, Erik J. Lundin, Reiner Giesler, Margareta Johansson, Jan Karlsson

**Affiliations:** 1 Department of Renewable Resources, University of Alberta, 116 St & 85 Ave, Edmonton, AB, CA T6G 2R3 Canada; 2 Climate Impacts Research Centre (CIRC), Department of Ecology and Environmental Science, Umeå University, SE-901 87, Umeå, Sweden; 3 Department of Physical Geography and Ecosystem Science, Lund University, Sölvegatan 12, 223 62 Lund, Sweden; 4 Swedish Polar Research Secretariat, Abisko Scientific Research Station, SE-981 07, Abisko, Sweden

## Abstract

Northern regions have received considerable attention not only because the effects of climate change are amplified at high latitudes but also because this region holds vast amounts of carbon (C) stored in permafrost. These carbon stocks are vulnerable to warming temperatures and increased permafrost thaw and the breakdown and release of soil C in the form of carbon dioxide (CO_2_) and methane (CH_4_). The majority of research has focused on quantifying and upscaling the effects of thaw on CO_2_ and CH_4_ emissions from terrestrial systems. However, small ponds formed in permafrost wetlands following thawing have been recognized as hotspots for C emissions. Here, we examined the importance of small ponds for C fluxes in two permafrost wetland ecosystems in northern Sweden. Detailed flux estimates of thaw ponds during the growing season show that ponds emit, on average (±SD), 279 ± 415 and 7 ± 11 mmol C m^−2^ d^−1^ of CO_2_ and CH_4_, respectively. Importantly, addition of pond emissions to the total C budget of the wetland decreases the C sink by ~39%. Our results emphasize the need for integrated research linking C cycling on land and in water in order to make correct assessments of contemporary C balances.

## Introduction

Climate warming accelerates permafrost thaw in northern regions, leading to the breakdown and release of soil carbon (C) in the form of carbon dioxide (CO_2_) and methane (CH_4_)^1^. Northern permafrost wetlands are of particular importance due to their vast C stocks^2^, which are vulnerable to changes in climate^3^. Increases in ambient air temperatures will result in permafrost degradation and the release of stored C as CO_2_ and CH_4_, thus contributing to a positive feedback on the climate^1^. Degradation of permafrost and the ground slumping that follows can often cause shifts in vegetation composition and expansion of wet areas^4,5^ that may lead to the formation and growth of thaw ponds^6,7^. Thaw lakes and ponds have been suggested to play a particularly significant role in the permafrost C feedback loop by acting as direct conduits for the release of CO_2_ and CH_4_ to the atmosphere^1,8,9^. However, few in-depth pond studies exist, especially so regarding their quantitative importance for the C exchange between land and atmosphere. Freshwater lakes are well known to be significant emitters of CO_2_ and CH_4_ on a landscape scale^10–12^, but previous studies do not include empirical measurements from small thaw ponds. Here, we examined the importance of small ponds for C fluxes in permafrost wetlands in northern Sweden.

On a few occasions when C exchange between water and air has been measured in thaw ponds, they are frequently found to be high emitters of CO_2_ and CH_4_^7,13–15^. The importance of thaw ponds, however, is largely overlooked in large scale assessments of C cycling due to their small size and the difficulties in mapping them using satellite imagery. For example, most upscaling studies exclude ponds smaller than 0.001 km^2 ^^16^. Integrated studies in which direct C fluxes from small ponds in permafrost regions are included into a catchment-scale C balance are thus still lacking. This study is the first to directly include C exchange from small thaw ponds in a wetland C exchange estimate.

**Figure 1:**
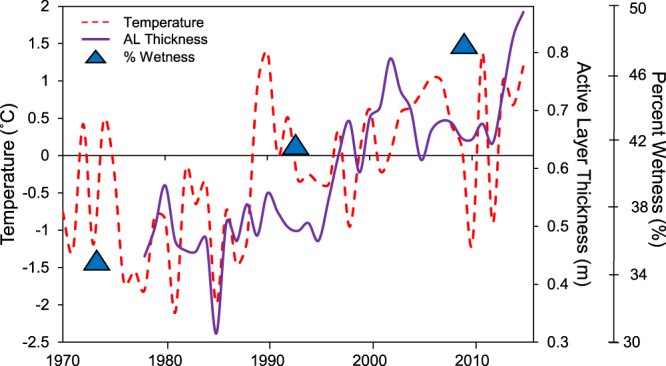
Decadal trends show increasing annual means for the Abisko area including annual air temperature (updated from Callaghan *et al*.^31^), autumnal active layer thickness (from 1978, updated from Akerman & Johansson^17^; bars represent standard deviations) and wetness data from Stordalen (based on three values from 1970, 1990 and 2008, updated from Malmer *et al*.^18^). Temperature data were collected at the Abisko Scientific Research Station, 10 km from Stordalen. Active layer thickness were measured in multiple mires in the Abisko area. The data on percent wetness from 2008 and ALT from 2007 is a contribution from this study.

In this study, we quantified atmospheric C exchange from 52 small thaw ponds (surface area 4–150 m^2^) in two permafrost wetlands to determine their role in the total (terrestrial + aquatic) net carbon balance (NCB). Of the 52 ponds, we carried out high-resolution chamber measurements of CO_2_ and CH_4_ fluxes over the full growing season of 15 ponds with different vegetation composition, shoreline erosion, and water sources (for map of sampling locations see Supplementary Figure S1). Furthermore, we measured dissolved CO_2_ and CH_4_ of the other 37 thaw ponds to assess large-scale spatial patterns. The study location in northern Sweden is a representative of regions with high permafrost vulnerability. The study region is characterized by discontinuous permafrost that has been experiencing substantial thawing during the last decades, mainly attributed to changes in air temperature and precipitation^17^ (Fig. 1). Detailed assessments of one of the wetlands have observed an increased wetness between 1970 and 2000^18^. By using aerial color imagery from 2008, we found that this trend has persisted alongside increases in annual air temperature and active layer thickness (Fig. 1). This change emphasizes the need to better quantify the role of ponds in the landscape C exchange.

## Results and Discussion

The results show strong oversaturation and high exchange of CO_2_ and CH_4_ to the atmosphere from the thaw ponds. The concentrations (mean ± standard error) of CO_2_ (467 ± 21 μM, n = 52) and CH_4_ (24 ± 3 μM, n = 52) are approximately an order of magnitude higher than the mean values reported for thaw ponds in northern Canada^4^ (means = 34 and 2 μM for CO_2_ and CH_4_, respectively), but are in the same range as permafrost ponds in west-central Siberia^19^ (range = 70–770 and 0.6–48 μM for CO_2_ and CH_4_, respectively). The average CO_2_ exchange measured from the ponds in our study (279 ± 58 mmol C m^−2^ d^−1^) was greater than exchanges (max = 114 mmol C m^−2^ d^−1^) reported from thaw ponds in northern Canada^7^ and from lakes (average value 15 mmol C m^−2^ d^−1^) in the same catchment as in this study^20^. The mean diffusive CH_4_ exchange (7 ± 0.2 mmol C m^−2^ d^−1^) in the ponds was among the highest diffusive CH_4_ fluxes reported, but was the same magnitude as found in ponds in eastern Siberia (average = 4 mmol C m^−2^ d^−1^)^14^. Ebullition was highly variable between lakes and accounted for 71% the total CH_4_ flux when observed (17 ± 6 mmol C m^−2^ d^−1^).

**Figure 2:**
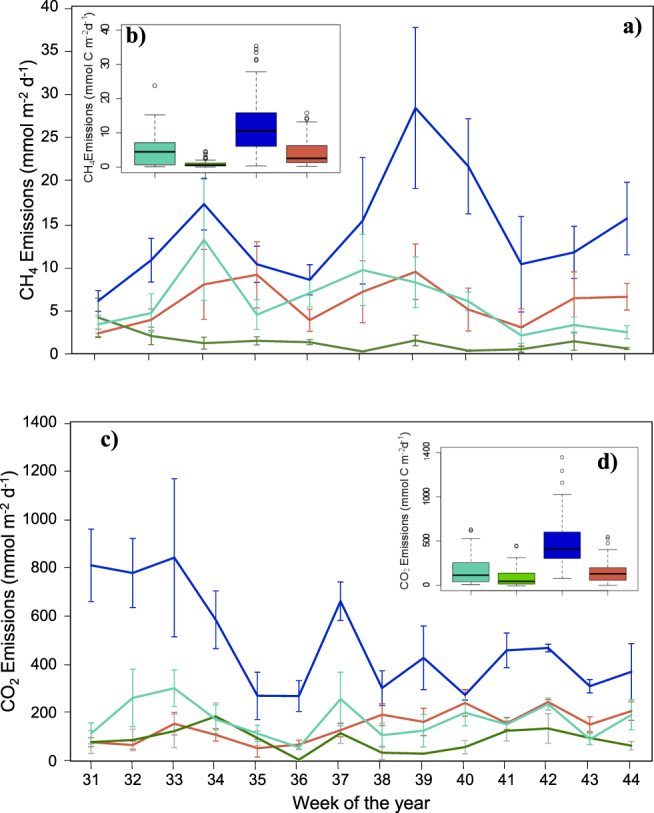
(**a**) Temporal, bi-weekly diffusive CH_4_ emissions for lake-fed (light green), moss-dominated (dark green), open-water (blue) and sedge-dominated (orange) ponds. (**b)** Mean CH_4_ emissions over the sampling period for each pond type. (**c**) Temporal, bi-weekly CO_2_ emissions. (**d**) Mean CO_2_ emissions over the sampling period for each pond type. Note the difference in scales. Error bars on bi-weekly plots represent the variation between mean fluxes from individual ponds within each pond type. Mean open-water pond emissions were the highest for both CO_2_ and CH_4_ compared to the rest of the pond types (Mann-Whitney U Statistical test, p-value < 0.001 for open-water and other pond type pairs).

Four pond types were identified during this study-including open water, moss-dominated, sedge-dominated, and lake-fed ponds (Supplementary Figure S2). Both CH_4_ and CO_2_ varied widely throughout the growing season and pond types showed different temporal variation. There were not significant differences in ebullition fluxes between pond types (Mann-Whitney U Statistical test, p-value = 0.44). Open-water systems had the highest mean diffusive emissions for both CO_2_ (610 ± 69 mmol C m^−2^ d^−1^) and CH_4_ (14 ± 2 mmol C m^−2^ d^−1^; Mann-Whitney U Statistical test, p-value < 0.001; Fig. 2b,d). These ponds had little to no vegetation at the surface or on the bottom and were surrounded by eroding banks, characteristics associated with high C exchange from ponds in northern Canada^7^. While this study was not designed to assess the importance of various drivers of C dynamics across ponds, the relatively high diffusive fluxes from open water ponds could be a result of high gas exchange velocities, relatively low photosynthetic CO_2_ fixation, and high terrestrial input of C and organic matter from adjacent thawing and eroding soils compared to vegetated ponds.

**Figure 3:**
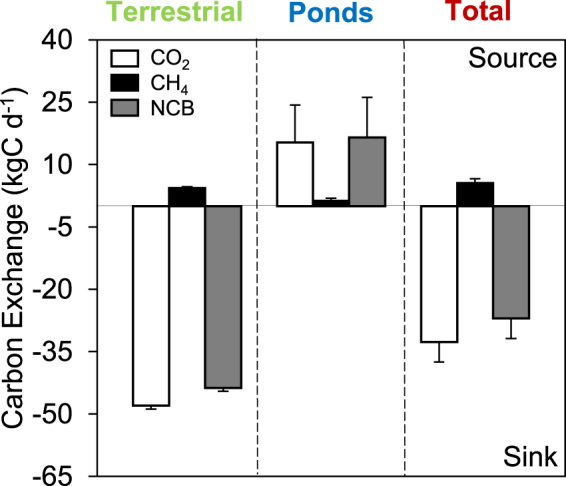
C exchange rate for CO_2_, CH_4_ (diffusion + ebullition), and CO_2_ + CH_4_ (NCB) for terrestrial wetland components, ponds, and the combination of terrestrial components and ponds over the growing season. The error bars for terrestrial components represent standard error in fluxes over a 7-year period^17^ (2002–2007), while the errors bars for ponds represent the uncertainty in areal coverage of ponds between 2000 and 2008.

A critical question is to what degree the emissions from thaw ponds are of quantitative importance compared to the overall C exchange from permafrost wetlands. One of the wetlands in this study (Stordalen) has been subject to extensive C exchange studies by chamber and eddy covariance techniques, enabling the first estimate of a complete NCB of a permafrost wetland and the specific importance of thaw ponds. Earlier studies in Stordalen found that the entire wetland is a large sink for CO_2_ and a source of CH_4_^21,22^. However, these studies focused mainly on the terrestrial or vegetated (non-open water patches) wetland and did not measure ponds directly. To incorporate the C emissions from ponds in our study into the NCB for the entire wetland, we combined our data with the mean terrestrial C exchange over the green season from 2002 to 2007 from Bäckstrand *et al*.^21^, and the areal coverage of open water ponds (0.43 ha) and the vegetated wetland (16.5 ha) in 2008. In the study by Bäckstrand *et al*.^22^, based on automated chamber measurements, terrestrial wetland components were divided into dry palsa, sphagnum, and wet/minerotrophic eriophorum patches. While their C-budget for the wetland includes wet areas, it does not include diffusive emission estimates for ponds specifically. The addition of the pond C emissions from 2015 to the NCB increased the CH_4_ source and decreased the CO_2_ sink (Fig. 3). The overall NCB, including both CO_2_-C and CH_4_-C, decreased from −44 kg C d^−1^ to −27 kg C d^−1^. We do note that C fluxes are dependent on multiple physical parameters and these parameters are not exactly the same between 2000 and 2015.

Although the C emission from the thaw ponds are of quantitative importance, the exact contribution should be treated with caution given the uncertainty in area of ponds. We estimated a range in contribution (Fig. 3) of thaw ponds to the NCB by using the area of ponds in 2000 (~1%)^18^ and 2008 (~2.5%). It is highly likely that the ponds now occupy an even larger percentage of the wetland landscape due to further permafrost thaw and saturated hollow development^23^, however images from this study’s research season were not available. Assuming pond developing continuous to follow the trend observed between 2000 and 2008 (1.5% increase), total pond area in 2015 can be estimated to be around 4%. At 4% of the total land cover, C emissions from ponds would reduce the NCB from ~−27 kgC d^−1^ to only ~−3 kgC d^−1^. Additionally, available images used for upscaling in this study only identify open-water ponds and do not distinguish vegetated ponds from the surrounding terrestrial vegetation; therefore, we only use open-water emission means in the upscaling estimate. Not including vegetated ponds is most likely a conservative estimate of the NCB. To upscale C emissions from small ponds and improve estimates of larger scale NCB of high latitude peatland regions, there is a need for higher resolution areal data and improved techniques to estimate the areal C contribution from different ponds in the landscape.

## Conclusion

**Figure 4:**
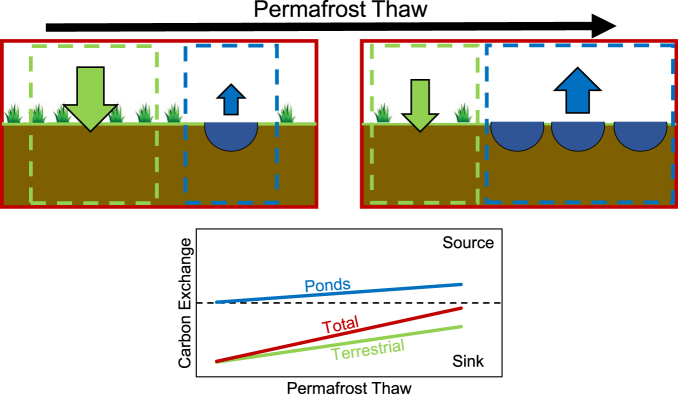
A conceptual diagram outlining the possible impact of permafrost thaw on the overall C sink capacity of northern wetlands where thawing results in increased coverage by ponds. The diagram emphasizes the need to integrate studies of terrestrial (green) and pond (blue) ecosystems in order to understand and project the carbon exchange of whole wetlands (red).

Our results suggest that ponds in northern permafrost wetlands are considerable sources of C to the atmosphere, significantly offsetting the net C uptake of wetlands, and thus reducing overall atmospheric C sink strength of the landscape. Thaw ponds cover a significant portion of land in the north^6^, and it is likely that those systems represent an important and sensitive component of the permafrost carbon feedback loop. The role of ponds as hotspots for C exchange following permafrost thaw and subsequent pond formation may be of increasing importance in permafrost regions in future decades following predicted permafrost thaw^6^. While projecting the trajectory of pond development and C exchange is highly uncertain, pond coverage may even decrease in some regions following thawing^24,25^, our results suggest that C release from thaw ponds following permafrost thaw may strongly contribute to a decrease in the overall C sink capacity of northern wetlands (Fig. 4). To reduce uncertainties, there is a need for more integrated research and further experiments or manipulations that focus on incorporated small ponds into regional NCB.

Importantly, the type of pond and the vegetation that it is comprised of may be a driving factor. If ponds develop into sedge-dominated or deeper, open-water systems, more C may be released to the atmosphere. Vegetation shifts in this direction have already been observed during naturally induced permafrost thaw^17,18^ and experimentally due to increased snow cover^26^. However, if there is a trend towards moss-dominated ponds, atmospheric C emissions could be substantially less. Understanding and quantifying the C emissions from the different types of ponds is crucial to predicting and modeling future emissions from permafrost wetlands subject to thawing since the ponds are constantly changing and evolving. Our results emphasize the need for integrated research linking C cycling on land and in water to make correct assessments of contemporary and future C balances.

## Methods

### Site description

Sampling took place in two wetland complexes over the duration of the growing season (June-October) in 2015. The Stordalen (68 N, 19 E) and Storflaket (68 N, 19 E) wetlands are located in the same catchment, approximately 3 km apart, in a discontinuous permafrost zone east of Abisko, Sweden. Stordalen covers ~15 km^2^ and Storflaket ~3 km^2^. The wetland complexes contain raised patches of peat underlain by permafrost, which are surrounded by saturated hallows and ponds. The hollows are comprised of graminoids (Carex sp., Eriophorum sp.) and sphagnum sp., while the palsas are covered by woody shrubs (eg Betula nana), evergreen shrubs (eg, Andromeda polifolia, Empetrum hermaphroditum), moss (Sphagnum sp.) and lichen. The active layer thickness is approximately 0.5 m in the palsas and 1–3 meters in the hallows at both sites^17^. Yearly temperature and precipitation means for this region were recorded at the nearby Abisko Scientific Research Station (http://www.polar.se/abisko).

### Ponds

In total, we sampled 52 ponds. Six ponds in Storflaket and nine ponds in Stordalen were sampled twice a week from the end of June until ice cover in early October. An additional 37 ponds were sampled in July. The ponds were chosen to cover a variety of morphologies, vegetation types, and degrees of erosion along the shoreline. Ponds were classified into moss dominated, sedge dominated and open water based on within-pond (1 × 1 meter grid) surface vegetation composition using a cluster analyses (R Company 3.2.1, pvclust Package). We also separated lake fed ponds that occasionally received external inputs of water from nearby lake.

### Water sampling and analysis

Water temperature, dissolved oxygen, and barometric pressure were measured *in situ* four times a week using a YSI Professional Optical Dissolved Oxygen instrument (Yellow Springs Instruments, Ohio, USA), which was calibrated at the start of each week. Surface water samples were collected weekly in 0.5 L containers, pre-rinsed in the field with pond water. Samples were filtered the same day in the laboratory (Sarstedt Filtropur S 0.45 mm), acidified (DOC) thereafter refrigerated (DOC) until processing. Conductivity and pH were analyzed in the laboratory on the same day as sampling (MP220, Mettler Toledo International Inc.). Dissolved inorganic carbon (DIC) and dissolved CH_4_ samples were collected from the surface of the water twice a week using a five mL plastic syringe. Using a headspace method, 4 mL of water was injected into a pre-prepared, 22 mL vial (Wheaton) with 20 uL of 20% HCL^27^. Vials were stored in an upside down position at room temperature until analysis. Methane and DIC concentrations were determined using a gas chromatograph with a methanizer and flame-ionization detector (Clarus 500, Perkin Elmer Inc. U.S.). Internal standards were prepared with each batch of samples to correct for possible leakage of gas over time. Filtered DOC samples were run on a Shimadzu TOC-V CPH analyzer (Shimadzu Corporation, Japan).

Dissolved CO_2_ concentrations were measured twice a week using a Vaisala sensor attached to a handheld logger (Helsinki, Finland, GMM220 and GM70 series). Sensors were deployed at the water’s surface and were occasionally stirred until the concentration hit equilibrium (5–30 minutes). Concentrations were later back calibrated in the laboratory to correct for temperature and pressure settings^28^.

### Carbon dioxide fluxes

Carbon dioxide fluxes were measured twice a week at seven of the fifteen ponds using a floating chamber (volume = 10.9 L). A plastic chamber was equipped with a CO_2_ sensor and attached by rope to a pole and handheld data logger (Vaisala Inc. Helsinki, Finland, GMM220 and GM70 series). The chamber was floated on the surface of the water between 9 am and 3 pm for 5–10 minutes at each pond while the concentration was recorded. Using the linear change in concentration and the dimensions of the chamber, the flux (F) was estimated using equation 1:

1 $$F=\frac{Vch\times S\times Kh\times P\times Q}{A}$$

where *Vch* is the volume of the chamber (m^3^), *A* is the area of the chamber (m^2^), *S* is the linear change in concentration measured over the five-minute period (ppm/min), *Kh* is Henry’s law constant (dependent on temperature), *P* is the pressure (atm), and *Q* is a conversion factor to set the flux units to mmol Cm^−2^ d^−1^. To calculate the flux of CO_2_ from the ponds that were not directly measured with a chamber, we used the concentration of dissolved CO_2_ and a variation of Fick’s law (eq. 2) to determine the piston velocity coefficient (k):

2 $$F=k(Cw-Ca)$$

which can be rearranged to solve for k as:

3 $$k=\frac{F}{(Cw-Ca)}$$

where F is the chamber flux (umol Cm^−2^ d^−1^), Cw is the concentration of dissolved gas at equilibrium with the headspace (umol Cm^−3^) and Ca is the concentration of CO_2_ in the atmosphere. All dissolved gas concentrations (CO_2_ and CH_4_) were calculated using Henry’s law:

4 $$Ga{s}_{aq}=Kh\times pGAS$$

where *pGAS* is the partial pressure of the gas in the headspace (ppm). The calculated *k* for both CO_2_ and CH_4_ values were normalized to 20 °C by calculating *k*_600_ values^29^.

### Methane fluxes

A 24-hour static chamber (volume = 10.9 L) flux method was used to measure total CH_4_ emissions at each pond^30^. Flux measurements were taken on a biweekly schedule. To separate the contributions of ebullition events from the diffusive flux, we looked at the distribution of the experimentally derived *k*_600_ values from the CO_2_ chamber flux measurements. Measured CH_4_ fluxes with *k*_600_ values above the mean CO_2_ *k*_600_ value (0.79 m d^−1^) were not considered in the diffusive flux calculations. A total of 22% (61 of 280) measurements were determined to be effected by ebullition (based on *k*_600_ > 0.79 m d^−1^) and ebullition were estimated by subtracting the average diffusive flux from the total flux of ebullition-impacted chambers^30^. Given that ebullition is highly sporadic and spatially heterogeneous, we reported ebullition as a mean rate.

### Statistics

Statistical analyses were done using the R statistics package (3.2.2, The R Company). Both CO_2_ and CH_4_ fluxes were normally distributed, however, the variances and sample sizes between pond types and monthly fluxes were not the same, therefore, we used the Kruskal-Wallis test for multiple group analyses and the Mann-Whitney *U* tests for follow-up pair-wise comparisons (p < 0.05). *K*_600_ values for CO_2_ and CH_4_ showed similar distributions.

### GIS Analysis and Carbon Budget Estimate

Pond area within Stordalen was calculated using ArcGIS software (esri. ArcGIS Desktop. 10.1 Redlands, CA). We used a mosaic of real-color images (8 × 8 cm resolution) of Stordalen mire taken in August 2008. The mosaic was clipped to represent the same area as presented in Malmer *et al*.^18^ and as used in Bäckstrand *et al*.^21^. Then, digital classification of open water ponds was determined with the help of user-selected training locations. Once training sites were selected, we used supervised classification to identify all open water ponds in the mire. We then used the pixel count of all areas classified as a pond to determine the total pond area in the mire.

To incorporate C emissions from ponds into the terrestrial NCB for the wetland, we used pond flux measurements from Storflaket and Stordalen and terrestrial flux values reported in Bäckstrand *et al*.^21^. In this study, the wetland was split into dry palsa, sphagnum, and wet/minerotrophic eriophorum components. Fluxes were measured from these patch types over a period of 7 years. The average emission rates from each land-cover type and the average number of growing days over the 7-year period were used in our estimate of total C loss and uptake from the terrestrial components of the mire. Importantly, while Bäckstrand *et al*.’s C-budget for the wetland includes wet areas, it does not include fluxes from open patches of water. Here we included both diffusive and ebullition fluxes in the previous NBC estimate by multiplying the mean daily flux from our study for both CO_2_ and CH_4_ from open-water ponds with the total pond area of the wetland. Total CO_2_ and CH_4_ emissions over the growing season (averaged from the previous 7 year data set) from the ponds were then added to total C emission and uptake estimates for the terrestrial components of the mire. Only fluxes and areas of open water ponds were included in upscaling due to difficulties associated with separating moss and sedge dominated ponds from wet, minerotrophic patches in the aerial image.

### Data availability

Data are available upon request.

## Electronic supplementary material

Supplementary Information

## References

[1] Schuur EAG (2008). Vulnerability of Permafrost Carbon to Climate Change: Implications for the Global Carbon Cycle. BioScience.

[2] Turunen J, Tomppo E, Tolonen K, Reinikainen A (2002). Estimating carbon accumulation rates of undrained mires in Finland – application to boreal and subarctic regions. The Holocene.

[3] Fronzek S, Luoto M, Carter T (2006). Potential effect of climate change on the distribution of palsa mires in subarctic Fennoscandia. Climate Research.

[4] McLaughlin J, Webster K (2014). Effects of Climate Change on Peatlands in the Far North of Ontario, Canada: a Synthesis. Arctic, Antarctic, and Alpine Research.

[5] Johansson C, Pohjola VA, Jonasson C, Callaghan TV (2011). Multi-Decadal Changes in Snow Characteristics in Sub-Arctic Sweden. AMBIO.

[6] Olefeldt D (2016). Circumpolar distribution and carbon storage of thermokarst landscapes. Nat Comms.

[7] Laurion I (2009). Variability in greenhouse gas emissions from permafrost thaw ponds. Limnol. Oceanogr., 55(1), 2010, 115–133. Limnol. Oceanogr..

[8] Walter, K. M., Chanton, J. P., Chapin, F. S. I., Schuur, E. A. G. & Zimov, S. A. Methane production and bubble emissions from arctic lakes: Isotopic implications for source pathways and ages. *J. Geophys. Res. Biogeosci*. **113** (2008).

[9] Holgerson MA, Raymond PA (2015). Drivers of carbon dioxide and methane supersaturation in small, temporary ponds. Biogeochemistry.

[10] Drake TW, Raymond PA, Spencer RGM (2017). Terrestrial carbon inputs to inland waters: A current synthesis of estimates and uncertainty. Limnol. Oceanogr..

[11] Cole JJ (2007). Plumbing the Global Carbon Cycle: Integrating Inland Waters into the Terrestrial Carbon Budget. Ecosystems.

[12] Kling GW, Kipphut GW, Miller MC (1992). The flux of CO2 and CH4 from lakes and rivers in arctic Alaska. Hydrobiologia.

[13] Wik M, Varner RK, Anthony KW, MacIntyre S, Bastviken D (2016). Climate-sensitive northern lakes and ponds are critical components of methane release. Nature Geosci.

[14] Knoblauch C, Spott O, Evgrafova S, Kutzbach L, Pfeiffer E-M (2016). Regulation of methane production, oxidation, and emission by vascular plants and bryophytes in ponds of the northeast Siberian polygonal tundra. J. Geophys. Res..

[15] Liebner S (2011). Methane oxidation associated with submerged brown mosses reduces methane emissions from Siberian polygonal tundra. Journal of Ecology.

[16] Holgerson MA, Raymond PA (2016). Large contribution to inland water CO2 and CH4 emissions from very small ponds. Nature Geosci.

[17] Åkerman HJ, Johansson M (2008). Thawing permafrost and thicker active layers in sub-arctic Sweden. Permafrost Periglac. Process..

[18] Malmer. (2006). Decadal vegetation changes in a northern peatland, greenhouse gas fluxes and net radiative forcing. Glob Change Biol.

[19] Pokrovsky OS, Shirokova LS, Kirpotin SN, Kulizhsky SP, Vorobiev SN (2013). Impact of western Siberia heat wave 2012 on greenhouse gases and trace metal concentration in thaw lakes of discontinuous permafrost zone. Biogeosciences.

[20] Lundin EJ (2016). Is the subarctic landscape still a carbon sink? Evidence from a detailed catchment balance. J. Geophys. Res..

[21] Bäckstrand K (2010). Annual carbon gas budget for a subarctic peatland, Northern Sweden. Biogeosciences.

[22] Christensen TR (2012). Monitoring the Multi-Year Carbon Balance of a Subarctic Palsa Mire with Micrometeorological Techniques. AMBIO.

[23] Stiegler, C. Surface energy exchange and land-atmosphere interactions of Arctic and subarctic tundra ecosystems under climate change. 1–56 (2016).

[24] Smith LC, Sheng Y, MacDonald GM, Hinzman LD (2005). Disappearing Arctic Lakes. Science.

[25] Andresen CG, Lougheed VL (2015). Disappearing Arctic tundra ponds: Fine-scale analysis of surface hydrology in drained thaw lake basins over a 65 year period (1948–2013). J. Geophys. Res. Biogeosci..

[26] Johansson M (2013). Rapid responses of permafrost and vegetation to experimentally increased snow cover in sub-arctic Sweden. Environ. Res. Lett..

[27] Karlsson J (2010). Quantifying the relative importance of lake emissions in the carbon budget of a subarctic catchment. J. Geophys. Res..

[28] Johnson, M. S. *et al*. Direct and continuous measurement of dissolved carbon dioxide in freshwater aquatic systems-method and applications. *Ecohydrol*. n/a–n/a, 10.1002/eco.95 (2009).

[29] Jahne B, Heinz G, Dietrich W (1987). Measurement of the diffusion coefficients of sparingly soluble gases in water. J. Geophys. Res..

[30] Bastviken, D., Cole, J., Pace, M. & Tranvik, L. Methane emissions from lakes: Dependence of lake characteristics, two regional assessments, and a global estimate. *Global Biogeochem. Cycles* **18**, n/a–n/a (2004).

[31] Callaghan, T. V. *et al*. A new climate era in the sub-Arctic: Accelerating climate changes and multiple impacts. *Geophys. Res. Lett*. **37**, n/a–n/a (2010).

