# Supplementary material for: Emissions from thaw ponds largely offset the carbon sink of northern permafrost wetlands
Source: Sci Rep. 2018 Jun 22;8:9535. doi: 10.1038/s41598-018-27770-x (PMC6015042; doi:10.1038/s41598-018-27770-x)
Supplement: Supplementary file 1 — Supplementary Information [file 41598_2018_27770_MOESM1_ESM.docx]

**Supplementary Information**

**Emissions from thaw ponds largely offset the carbon sink of northern permafrost wetlands**

*McKenzie Kuhn^1,2^, Erik J. Lundin^2,4^, Reiner Giesler^2^, Margareta Johansson^3^, Jan Karlsson^2^

1. Department of Renewable Resources, University of Alberta, 116 St & 85 Ave, Edmonton, AB, CA T6G 2R3, email: mckenzie@ualberta.ca

2. Climate Impacts Research Centre (CIRC), Department of Ecology and Environmental Science, Umeå University, SE-901 87 Umeå, Sweden

3. Department of Physical Geography and Ecosystem Science, Lund University, Sölvegatan 12, 223 62 Lund, Sweden

4. Swedish Polar Research Secretariat, Abisko Scientific Station, SE-981 07 Abisko, Sweden

**
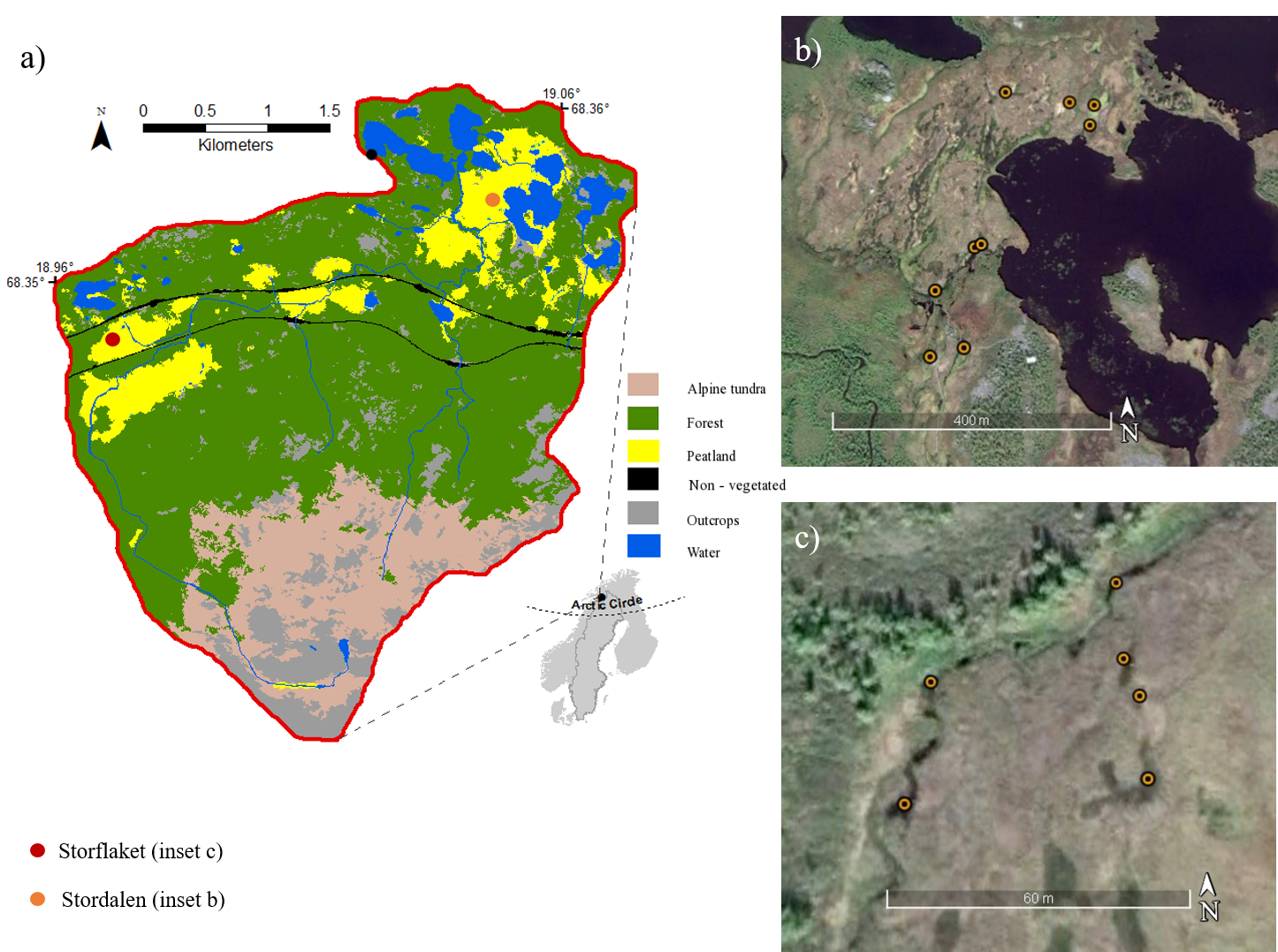
**

Figure S1: Pond sampling locations. a) A map of the greater Stordalen and Storflaket catchment area in northern Sweden. b) Location of the 9 detailed sampled ponds in Stordalen. c) Location of the 6 detailed sampled ponds in Storflaket. Map adapted from Lundin et al. 2015.

**
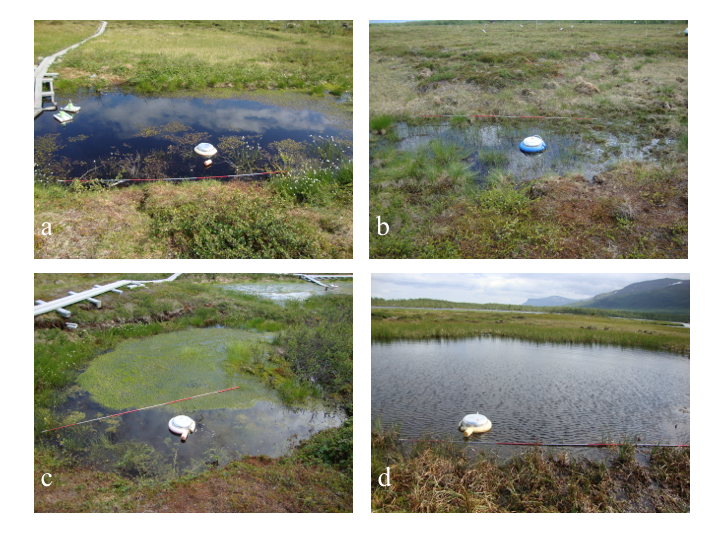
**

Figure S2: Examples of a) open-water, b) sedge-dominated, c) moss-dominated ponds, and d) lake-fed pond types. Also pictured are plastic floating chambers used to measure gas flux and a 2-meter measuring stick for scale.

Supplementary Information References

Lundin, E. J. *et al.* Large difference in carbon emission-burial balances between boreal and arctic lakes. *Sci. Rep.* **5**, 14248; doi: 10.1038/srep14248 (2015).
